# Analysis of dynamic networks based on the Ising model for the case of study of co-authorship of scientific articles

**DOI:** 10.1038/s41598-021-85041-8

**Published:** 2021-03-11

**Authors:** V. Andrea Hurtado-Marín, J. Dario Agudelo-Giraldo, Sebastian Robledo, Elisabeth Restrepo-Parra

**Affiliations:** 1 PCM Computational Applications, Universidad Nacional de Colombia, Manizales, Colombia; 2 Departamento de Física y Matemáticas, Universidad Autónoma Manizales, Manizales, Colombia; 3 ECOSOL Research Group, Universidad Católica Luis Amigó, Manizales, Colombia; 4 Centro de Bioinformática y Biología Computacional de Colombia, BIOS, Ecoparque Los Yarumos, Manizales, Colombia

**Keywords:** Complex networks, Statistical physics, Social evolution

## Abstract

Two computational methods based on the Ising model were implemented for studying temporal dynamic in co-authorship networks: an interpretative for real networks and another for simulation via Monte Carlo. The objective of simulation networks is to evaluate if the Ising model describes in similar way the dynamic of the network and of the magnetic system, so that it can be found a generalized explanation to the behaviours observed in real networks. The scientific papers used for building the real networks were acquired from WoS core collection. The variables for each record took into account bibliographic references. The search equation for each network considered specific topics trying to obtain an advanced temporal evolution in terms of the addition of new nodes; that means 3 steps, a time to reach the interest of the scientific community, a gradual increase until reaching a peak and finally, a decreasing trend by losing of novelty. It is possible to conclude that both methods are consistent with each other, showing that the Ising model can predict behaviours such as the number and size of communities (or domains) according to the temporal distribution of new nodes.

## Introduction

The modeling of social phenomena with physical bases has allowed an affirmative answer to whether there are or not laws that govern them^1^. The concept has gained strength in recent years thanks to two crucial facts: on the one hand, the access to large databases, turning society into a huge sample for research^2^; and on the other, to advances in the study and characterization of complex networks^3^. In principle, it has been assumed that social groups are systems composed of interacting agents capable of taking a stance in the face of an external disturbance, in a similar way to a many-body system in statistical physics^4–6^. Different types of algorithms have been proposed to detect communities in the framework of physics, Potts model in^7^ and label propagation algorithm (LPA)^8,9^ are some examples. In particular, algorithms under Ising model also has been widely implemented^6,10,11^.

One of the most significant current discussions in social networks is understanding their dynamics through the time. For instance in^12^, it has been shown that the addition of the social dynamics decreases the network influence on the decision of their members, and in^13^ is highlighted the importance of considering the collaboration dynamic among academics to understand the evolution of a research topic. Therefore, the metrics of temporal evolution are the crucial mechanisms behind it. Trying to solve this problem, in this work is proposed the Ising model for detecting evolution patterns in social communities.

Under the precept that by their nature, there are different types of networks, and that they diverge in a large number of behaviors^14^, this research proposes to study co-authorship of scientific articles around specific topics or areas. A co-authorship network is made up of scientists with close social contacts whose interactions represent collaborations or cooperation^15–17^. Although in recent studies, these contacts have evidenced the formation of the scientific communities, most of the works developed lack dynamic analysis^6,15,18–21^, or explicitly despite the variability of weight in each link^1,22–24^. For this reason, one of the objectives of our study has been to look for predictable patterns or behaviors that can arise from these dynamic interactions. Specifically, the formation of scientific communities is analyzed taking into account the appearance and the redistribution of new nodes and the weight of the links.

The studies required a comparative analysis between the physical description of real networks and spins simulations. The objective of this work was to observe if the Ising model describes the temporal dynamic of real networks and from this give a generalized explanation of the behaviors observed by mean of simulation. The real networks procedure consisted of applied an improved version of the algorithm presented by Son et al. for flow networks^6^. According to a recent classification presented by El-Moussaqui et al. for identification of communities in complex networks^25^, this method has a divisive and hierarchical approach, reason by which is denominated here as DHM. For the magnetic simulations, the Ising model with mean field discrimination was implemented using the Monte Carlo method (MCM). The correspondence between the two methods is analyzed by observables such as internal energy and the number and size of communities (or domains).

Some other works have taken advantage of different models and computational tools for improving the analysis of collaborative networks. For instance, in^26^ is used a bipartite graph approach blended to joint in the structure as researchers attitudes and interests as the community’s recognition. In^27^ for exanimating disambiguating names in references based upon the topological and the hierarchical descriptions, in^28^ an analysis of patterns that relate ranking, first and last authors, specific roles, and others characteristics, indicate that must take into account the distinct types of scientific contributions, and in^29^ coauthors characteristics are obtained applying learning models with training.

## Methodology

As mentioned above, two developments were necessary to add to the analysis process: an experimental one, obtained from real co-authorship networks with the authors as nodes, and the other by simulation under equivalent interaction situations, but between spin moments. The procedure for each of them is described below:

### A. Ising model applied to the interpretation of co-authorship networks via DHM

In the construction of the network, each node represents an author and each link a scientific collaboration. The scientific papers were obtained from the Core Collection of Web of Science (WoS), one of the most relevant bibliographic databases widely used in academia^30,31^. Core collection consists of ten sub-datasets (eight citation indexes and two chemical indexes), with information from over 18,000 high impact journals, over 180,000 conference proceedings, and over 80,000 books^32^. Four co-authorship networks were built with the records of specific queries by topic and title, and limiting the document just to scientific articles. The research topics and title were: econophysics, magnetoresistance—nanoparticles, Monte Carlo—thin films, and Monte Carlo—core shell nanoparticles.

These four networks were selected from a large number of searches, three of them based upon our expertise in condensed matter physics. The condition of selection was an advanced temporal evolution in terms of the addition of new nodes. That means the presence of 3 steps: a time to reach the interest of the scientific community, a gradual increase until reaching a peak, and finally, a decreasing trend by losing of novelty. Temporal evolutions can be fitted with an extreme value distribution presented by Eq. (1) (as is present in the “Results and discussion” section). In this distribution, *N*_*o*_ is a number that varies according to the total number of nodes (or spins), *t*_*p*_ is the time for which the function has a maximum and *β* is a scale parameter. It should be clarified at this point that dynamic networks can present different types of distribution for the entry of new nodes. For this reason, it was ensured that both the analysis of the real networks and the Monte Carlo simulation were characterized by the shape of distribution of new nodes.

1 $$N\left( {t_{s} } \right) = N_{o} *\frac{1}{\beta }{\text{exp}}\left( {\frac{{t_{s} - t_{p} }}{\beta }} \right){\text{exp}}\left( { - {\text{exp}}\left( {\frac{{t_{s} - t_{p} }}{\beta }} \right)} \right).$$

The variables for each record were: ID (assigned by WoS), DOI (digital object identifier), authors, year of publication and bibliographic references. For the articles present within the bibliographic references with DOI, Crossref was used to obtain the information of their authors and their year of publication. A minimum standard was set to label authors with the initials of the first name and the first surname (all written in capital letters), thus reducing the typographic differences between journals. The software for the construction and identification of communities of the co-authorship networks was compiled in R.

The networks built are dynamic (changing over time). For this reason, the year variable was transformed into time steps (*t*_*s*_). For each of the steps, a static network was built, taking into account the nodes and links that emerged in earlier times. Additionally, in the present work, the weight of the links was weighted based on the number of documents written by pairs. To identify the scientific communities, the procedure proposed in^6^ was used, which was applied to each of the connected components of the static network. Initially, the two most influential nodes within the network that generate a community structure are identified, denoted as *s* and *t*. Node *s* is the highest degree and node *t* is the next highest degree node with which this structure is generated. The algorithm to identify the communities is based on the existing analogy between a flow network and a ferromagnetic random field Ising model^33^ whose Hamiltonian is given by:

2 $$H = - \frac{1}{2}\mathop \sum \limits_{i,j = 1}^{n} J_{ij} \sigma_{i} \sigma_{j} - \mathop \sum \limits_{i = 1}^{n} B_{i} \sigma_{i} ,$$

where each node *i* represents a spin *σ*_*i*_ in the magnetic system, *J*_*ij*_ is the exchange constant between the spins and *B*_*i*_ is the field on the spin *σ*_*i*_. The exchange constant *J*_*ij*_ between two spins *σ*_*i*_ and *σ*_*j*_ is equal to the weight of the link. The weight is obtained by the number of interactions between two authors; that is to say, the number of scientific documents that were published together. For this reason, only ferromagnetic couplings (*J*_*ij*_ ≥ 0) were considered. The steps for each component are: (i) build a flow network^33^, (ii) calculating the maximum flow^34^ and (iii) identify the community structure. A flow network contains the same nodes and links as the original network plus two additional nodes called source and sink denoted as *s* and *t*. In the algorithm proposed by Son et al. in^6^, all the possibilities of pair of nodes *s* and *t* were tested. But the authors propose that these nodes could be identified from the nodes of higher degree, to reduce the computational cost.

The magnetic field distribution used is given by Eq. (3), for two nodes *s* and *t*; this is equivalent to imposing the boundary condition *σ*_*s*_ =  + 1 and *σ*_*t*_ =  − 1, which induces frustration in the system. In the flow network the weights in the links are given by *c*_*ij*_ = 2*J*_*ij*_^6^ (also denominated as capacity). The infinite value of the local field applied to the spins *σ*_*s*_ and *σ*_*t*_, within the computational simulations, was calculated as $B_{s} = \mathop \sum \limits_{i, j = 1}^{n} c_{ij}$ and $B_{t} = - \mathop \sum \limits_{i, j = 1}^{n} c_{ij}$, respectively.

3 $$B_{i} = \left\{ {\begin{array}{*{20}c} { + \infty ,} & {{\text{para}}} & {i = s} \\ { - \infty ,} & {{\text{para}}} & {i = t} \\ {0,} & {{\text{para}}} & {i \ne s,t} \\ \end{array} .} \right.$$

To identify the communities, the criterion established by the maximum flow—minimum cut theorem is used. The algorithm consists of subdividing the sample continuously until the division does not have a community structure. Each iterative subdivision always leads to identifying two sets *C*_*s*_ and *C*_*t*_, with the two most influential nodes. To generate a subdivision, it must be satisfied that $\frac{{\ln \left( {D_{st} } \right)}}{\ln \left( n \right)} > 1$, where *D*_*st*_ is the product between the number of nodes that belong to sets *C*_*s*_ and *C*_*t*_, and *n* is the number of internal nodes by subdivision. At the end iterative subdivisions, when $\frac{{\ln \left( {D_{st} } \right)}}{\ln \left( n \right)} < 1$, the set of *n* nodes correspond to a community. These point in the same direction of *s*. More information on this procedure can be found at^6,33^. The communities should have more than 2 nodes. Nodes that do not belong to a community have a spin randomly assigned. The local field value for each community is assigned according to *B*_*s*_ and *B*_*t*_.

### B. Ising model simulated via MCM

The computational program for the magnetic modeling via Monte Carlo was thought from preliminary results obtained for the co-authorship networks. The spins were randomly located within a box of length *L* = 16 muc, where muc are magnetic cell units (relative to positions in a cubic crystalline system). The minimum distance between moments was set at 1 muc. The addition of new moments to the system obeyed a location probability given by *P* = exp (0.5 (1 − *r*_*ij*_)), whereby comparing it with a random number, the positions closest to that of the last spin added were privileged.

Additionally, each simulation was divided into time steps to introduce new spins to the system, according to the distribution presented in Eq. (1), with *No* = 2458 (maximum number of spins) and *β* = 6. This is in accordance with the fact that new authors (nodes) appear in the co-authorship networks over time (dynamic network). At each time, the system was simulated under a canonical assembly (NVT) at 60 K. In the preliminary results, the temperature did not show a significant influence. The time variable ranged from 1 to 40 steps. The final-time density was established at 0.6 spins per cell.

The Metropolis algorithm was implemented to generate the state fluctuations. Periodic boundary conditions were not taken into account. The number of Monte Carlo steps was set at 100,000 with a cutoff for calculating observables at 50,000 per time step. FORTRAN95 was the software used to compile the code.

The Hamiltonian used in the realization of this model is defined by Eq. (4), where *J*_*ij*_ was established at ± 10 meV. A value of *r*_*cut*_ = 1.5 muc was selected because, in physics publications, the number of authors in each article is approximately 4 on average. Therefore, choosing a larger cut-off radius would involve other types of networks in which the interactions are more numerous. Interactions with neighbors that are less than 1.25 muc apart were considered ferromagnetic (*J*_*ij*_ > 0), while at further distances, the interactions were antiferromagnetic (*J*_*ij*_ < 0). The mean field per site (*B*_*mean*_,_*i*_) was used to identify domain boundaries. In particular, if − 1 < *B*_*mean*_,_*i*_ < 1, *σ*_*i*_ is considered a frustrated spin, without domain. This phenomenon is also observed in co-authorship networks in which there are authors who do not belong to a defined scientific community.

4 $$H = - \mathop \sum \limits_{<i,j>} J_{ij} \sigma_{i} \sigma_{j} .$$

## Results and discussion

Table 1 presents information on the search equation for each network. The first four columns are information on the date of the query, the real time period, the number of articles found and the number of references. The next three columns correspond to information extracted from each network: the transformation of the period into the number of time steps, the total number of nodes (*N*_*n*_) and the total number of links (*N*_*l*_).

**Table 1:** Search information for each network. WoS source.

Search equation	Date of query	Real time period	Number of papers	Number of references	Time steps	*N*_*n*_	*N*_*l*_
TITLE: (econophysics)	29/02/2020	1930–2019	102	1751	66	2282	5242
TITLE: (magnetoresistance) AND TITLE: (nanoparticles)	29/02/2020	1953–2019	68	1463	54	4514	20,955
TITLE: (monte carlo) AND TITLE: (thin films)	29/02/2020	1930–2019	211	3840	73	8221	31,726
TOPIC: (monte carlo) AND TITLE: (core shell nanoparticles)	11/03/2020	1950–2019	58	1351	57	3528	15,534

**Figure 1:**
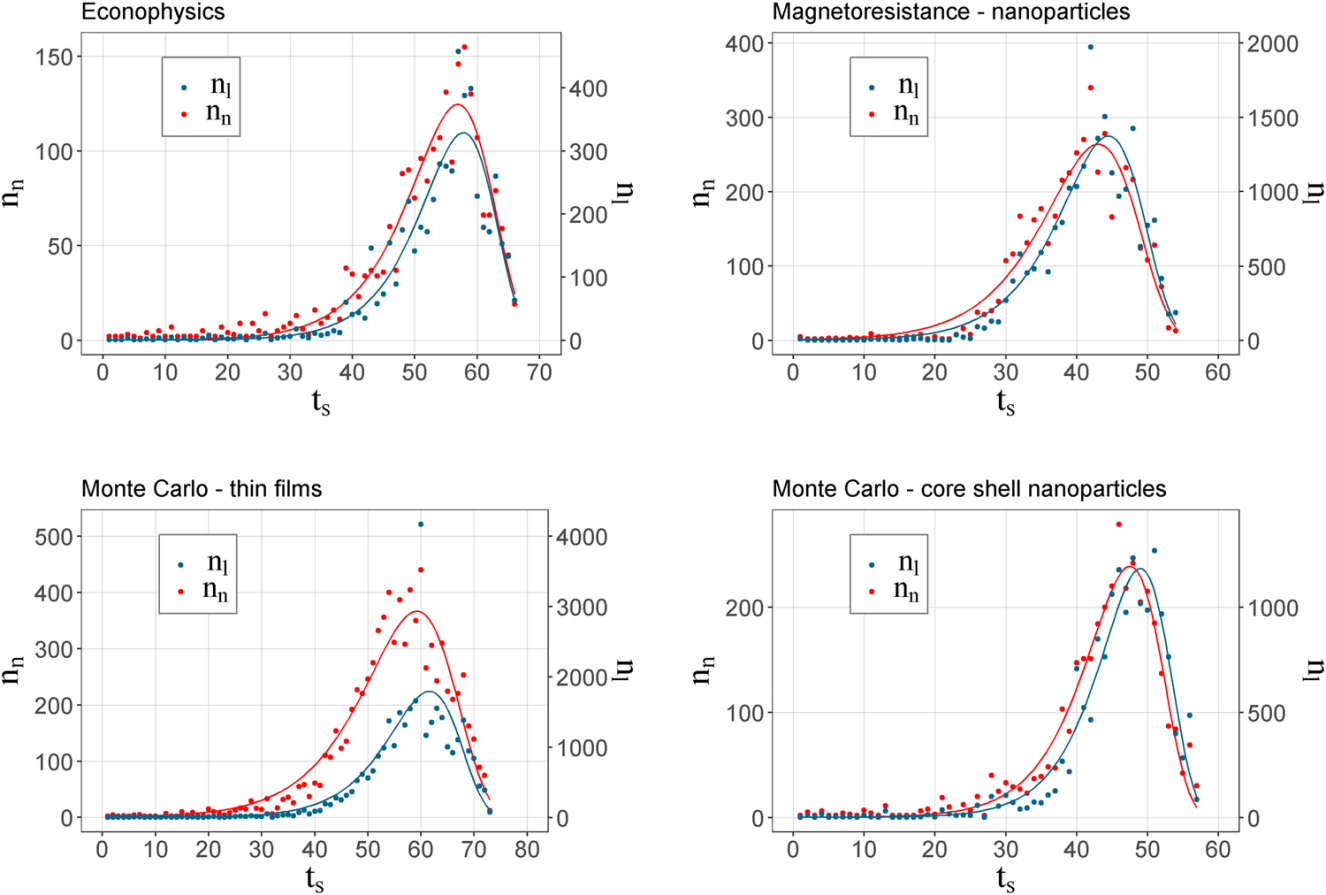
Number of new nodes and new links as a function of time for the different co-authorship networks.

**Figure 2:**
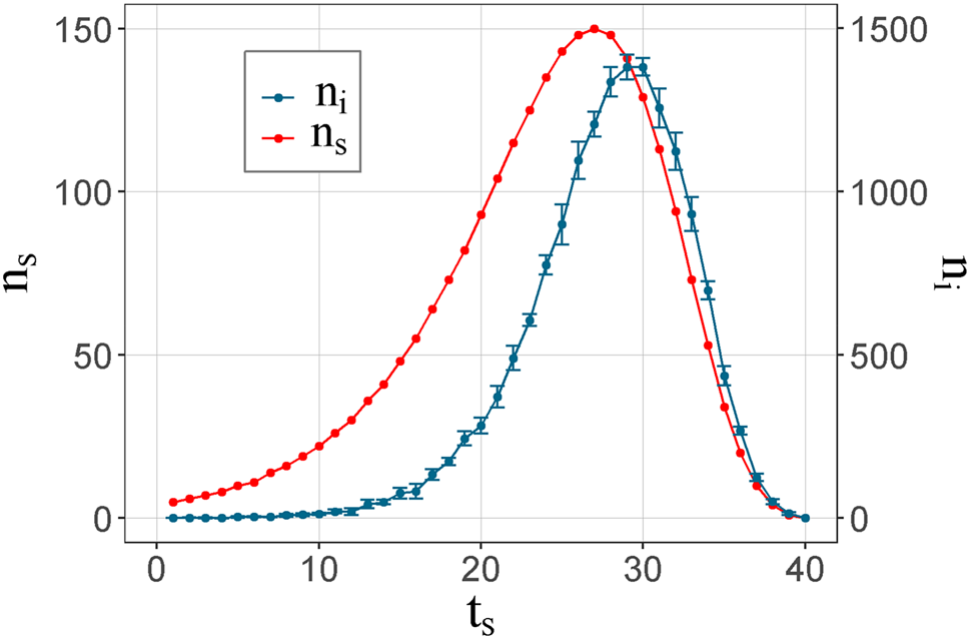
Temporal distribution of the insertion of new spins and the appearance of interactions for simulations with *t*_*p*_ = 27 in Monte Carlo method.

Figure 1 shows the temporal evolution of the number of new nodes and links for each network. As can be inferred, a new research topic takes time to reach the interest of the scientific community; later, there is an exponential increase in the number of new authors until reaching a peak time (*t*_*p*_) and, finally, the topic loses its appeal or novelty, showing a decreasing trend. Each of the curves is shown an adjustment to the distribution established by Eq. (1). This figure shows that networks first reach a maximum in new nodes than in new links. These results were also observed in the simulated behavior. Figure 2 presents the temporal distribution of new spins and new interactions for *t*_*p*_ = 27. As mentioned previously, the shape of the distribution is fixed with the same one with which the co-authorship networks were adjusted (Eq. (1)). The fact that the links come from a collective behavior causes any transition phase in them or in their observable derivatives to present this delay.

**Figure 3:**
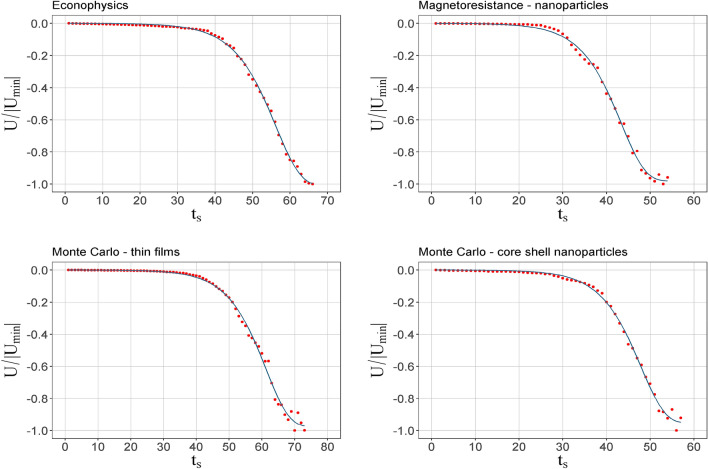
Normalized energy as a function of time for the co-authorship networks. The blue line is an adjustment to get the inflection point.

In Fig. 3, the energy (*U*) is presented as a function of time for the different networks. The values are normalized to the minimum value. *U* decreases with time due to the entry of new nodes whose interactions mostly contribute with negative interactions (Eq. (2)). The inflection point was obtained by fitting the exponential curve of Eq. (5), but decreasing. The fit is represented by a blue line on the graph. It is expected by the correspondence between energy and links, that these curves present a time at the inflection point, called *t*_*c*_, close to the *t*_*p*_ of the links. The values for *t*_*c*_, along with the peak times of nodes and links, are presented in Table 2. In the language of physics, this behavior is associates with the percolation threshold of the system^35^. Below the percolation threshold, the probability of finding a connected path between two separated spins in the system is very low; because of this reason, there cannot be any long-range magnetic ordering. Above the percolation threshold, the probability of finding connected paths increase, so exchange interaction broadcast further. In the language of networks, this inflection point means a transition from a state in which the system presents a fluctuating topology in terms of the distribution of nodes in the communities to a state with well-defined communities.

5 $$y = A*\left( {1 - {\text{exp}}\left( { - {\text{exp}}\left( {\frac{x - C}{B}} \right)} \right)} \right).$$

**Table 2:** Summary of characteristic times in co-authorship networks.

Search equation	Nodes *t*_*p*_	links *t*_*p*_	Energy *t*_*c*_	Number of communities, *t*_*c*_
TITLE: (econophysics)	56.9 ± 0.2	57.8 ± 0.3	56.2 ± 0.1	55.7 ± 0.2
TITLE: (magnetoresistance) AND TITLE: (nanoparticles)	43 ± 0.3	44.5 ± 0,3	43.4 ± 0,2	40.9 ± 0.2
TITLE: (monte carlo) AND TITLE: (thin films)	59.2 ± 0.3	61.6 ± 0.5	61.3 ± 0.2	55.3 ± 0.2
TOPIC: (monte carlo) AND TITLE: (core shell nanoparticles)	47.5 ± 0.2	49 ± 0.2	47.9 ± 0.2	45.2 ± 0.2

The difference between the times *t*_*c*_ of energy and *t*_*c*_ of the number of communities show that they are not the product of the same state transition.

**Figure 4:**
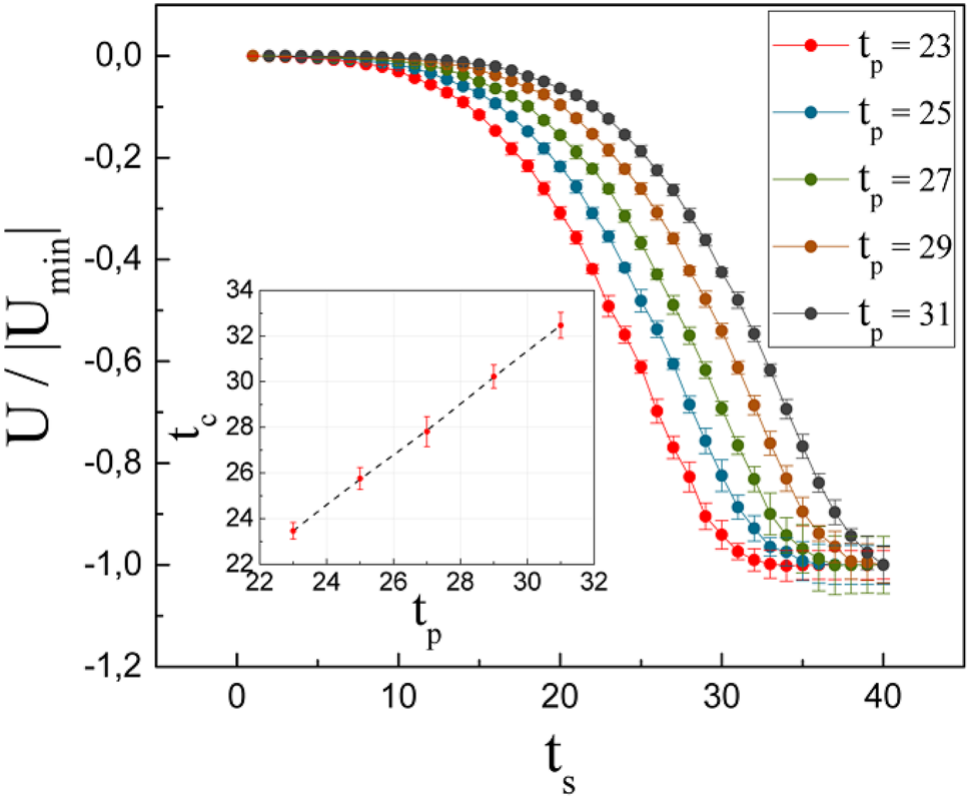
Normalized energy as a function of time for different values of *t*_*p*_. Inset: *t*_*c*_ as a function of *t*_*p*_.

To complement, Fig. 4 presents the energy as a function of time for different *t*_*p*_ in the spin distribution. The values are normalized to the minimum value. As in co-authorship networks, *U* decreases over time. This means that the number of interactions is mainly ferromagnetic, since there are a greater number of spins (nodes) in the domains (communities), than in the borders where antiferromagnetic interactions occur. *t*_*c*_ was found by fitting the curves to Eq. (6) and setting the second derivative equal to zero.

6 $$U = \frac{{ - {\text{arctan}}\left( {At_{s} + B} \right) + C}}{D}.$$

Since increasing *t*_*p*_ decreases the density of nodes and links in the first-time steps, it can be specified that the change of state given by *t*_*c*_ requires a minimum density. In the inset, in Fig. 4, it can be seen that a linear relationship can represent these two variables.

**Figure 5:**
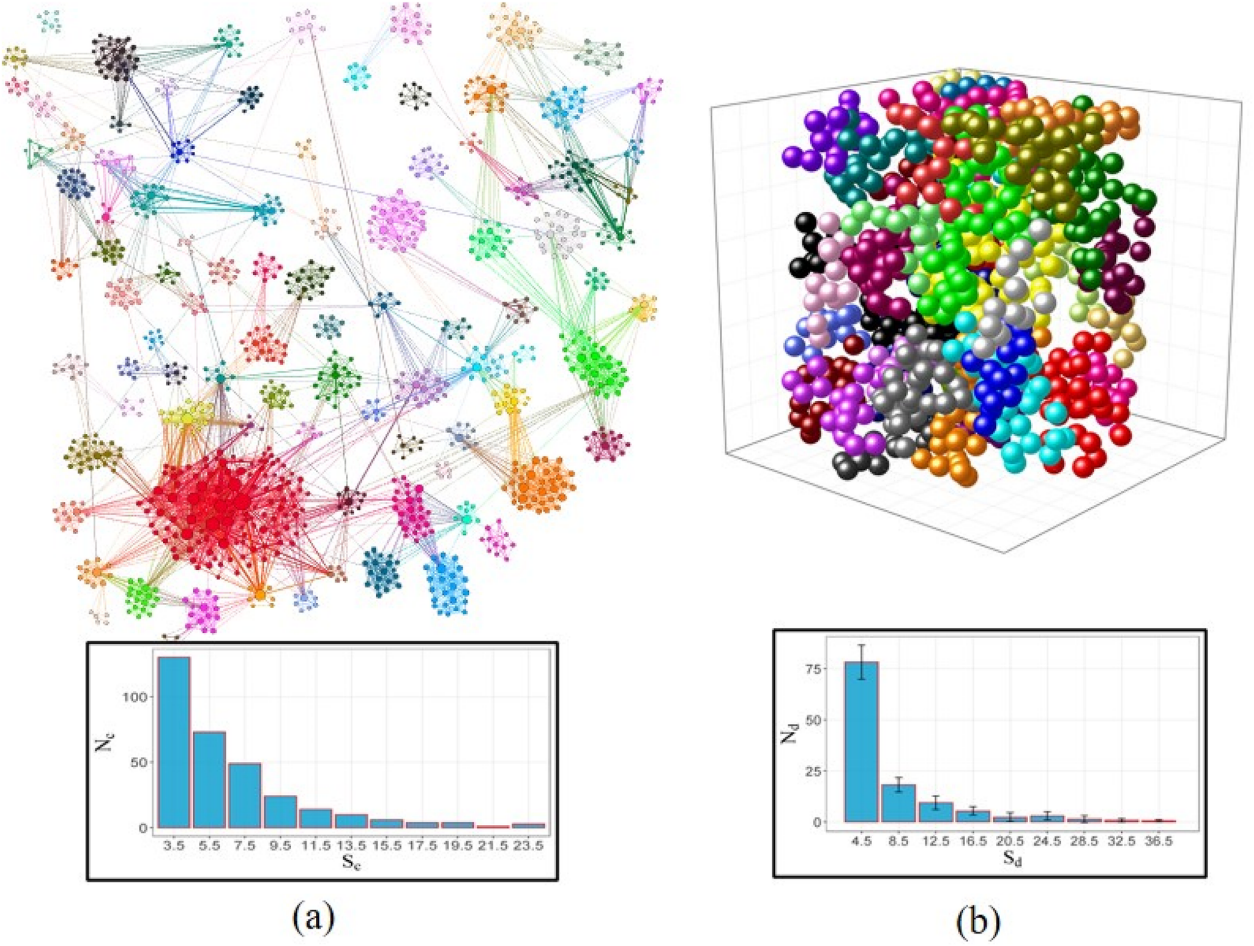
Images of (**a**) giant component of the core Shell nanoparticles network, showing the communities that have more than 4 nodes and (**b**) magnetic domains that have more than 10 spins, for a distribution of simulated atoms with *t*_*p*_ = 27. The histograms inserted in (**a**) and (**b**) represent the size distribution of the communities and domains, respectively.

Figure 5 presents descriptive images of the final samples and their communities (or domains), particularly in a) for the giant component of the core shell nanoparticles network with communities of more than 4 nodes, and b) for the distribution of simulated atoms for *t*_*p*_ = 27, there the domains that have more than 10 spins are shown. The internal histograms show the distribution of sizes, *S*_*c*_ and *S*_*d*_ for communities and domains, respectively.

It is remarkable to say that, at difference of other methods where a community is assigned to all nodes, even if it is not coherent, spin models are characterized because allows to determine the nodes that do not belong to a community. In the simulations, the boundaries of magnetic domains are formed from frustrated spins, which can be identified if mean field per site is low.

**Figure 6:**
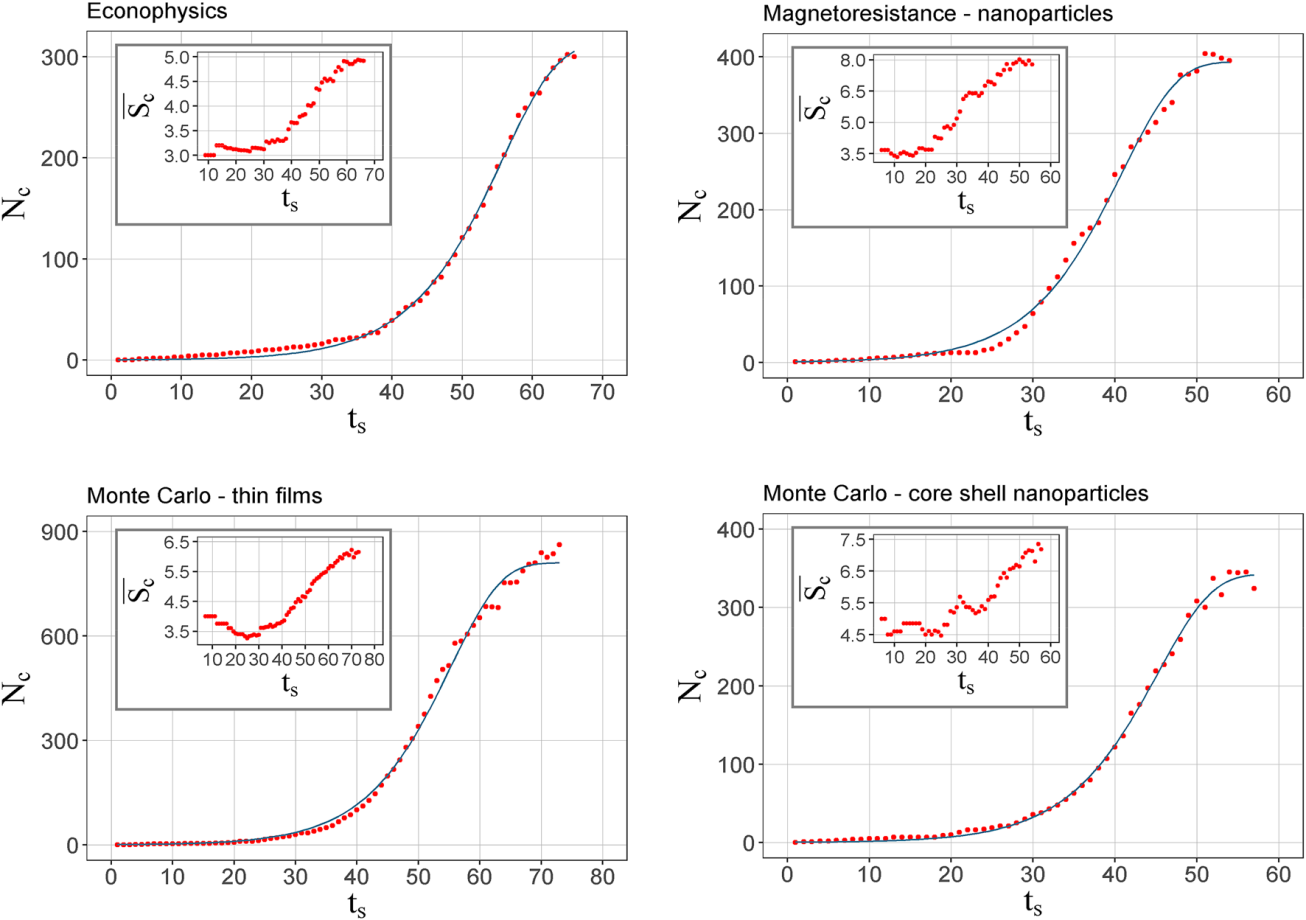
Number of communities as a function of time for the co-authorship networks. The inserted figures correspond to the temporal evolution of the average size of the community, respectively.

Figure 6 shows the number of communities (*N*_*c*_) as a function of time for the different networks. In the internal insets of each figure, the temporal evolution of the average community size ($\overline{{S_{c} }}$) in the number of nodes is presented. It was assumed that the formation of communities begins in the time in which at least 3 are observed. Given this condition of presence $\overline{{S_{c} }}$ begins to decline. This is likely because as nodes are added to the system, links are formed that favor the segmentation of the study. Then $\overline{{S_{c} }}$ increases by adding new nodes with the publication of new articles; however, over time the dominant effect is the formation of new communities of small sizes, as can be seen in the histogram in Fig. 5a. This previous analysis allows us to interpret the temporal curves of the number of communities. The adjustment was made to each of the trends using Eq. (5). In these curves, it can be observed that at first, there are no communities due to the publication of articles with only two authors at this time. Subsequently, there is a slow growth given the small number of new nodes. Then, the number of community experiences accelerated growth as a result of a higher rate of new nodes and collaborations. However, from the time step *t*_*c*_, at the inflection point, the growth rate is reduced. For each of the networks, the value for *t*_*c*_ is below the values of *t*_*p*_ for the new nodes and links (Table 2). In other words, the inflection point occurs before the system presents the largest number of new nodes and links. Additionally, the *t*_*c*_ taken from energy is also above that of the domains. Finally, in the last steps of time, *N*_*c*_ tends to stabilize due to the few authors that transit between communities in these times. In principle, the distribution established for the entry of new nodes could play a crucial role in predetermining the inflection point. This is very important, if that inflection point is known in real time, subsequent events can be predicted, including when the maximum number of new nodes will occur per year and an estimate of that number.

**Figure 7:**
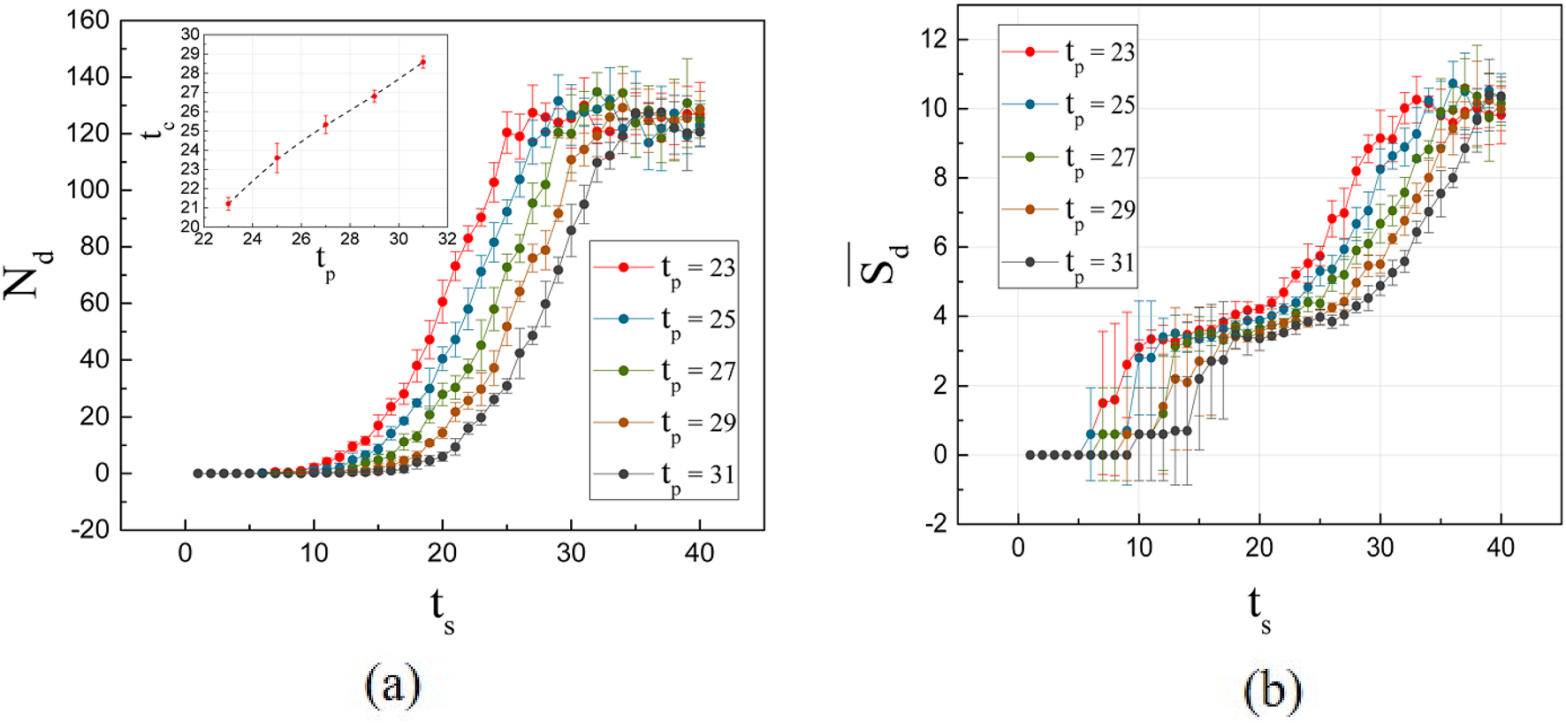
(**a**) Number of domains as a function of time for different values of *t*_*p*_ in Monte Carlo simulations. Inset: *t*_*c*_ as a function of *t*_*p*_, obtained from these trends. (**b**) Average domain size as a function of time for different values of *t*_*p*_.

The simulations also made it possible to analyze these trends in detail. Figure 7a presents the number of domains as a function of time for different *t*_*p*_ in the *n*_*s*_ distributions. Very similar trends are observed between one method and another, networks and simulation. In the first step the number of domains is zero due to the low number of spins and the competition dominated by temperature on a very low interaction energy in the system. As time passes, domains begin to form at high rates, more stable due to the increase in FM links, mainly. This process ends in an approximately constant value for *N*_*d*_ since, in the last steps, very few spins enter, actually affecting the system. Also, in Fig. 7a, the increase in *t*_*p*_ shows an increase in time at the inflection point. The relationship can be approximated to linearity, as seen in the inset figure. These times show to be lower than those observed for *t*_*c*_ in the energy and in *t*_*p*_ both for the nodes and the links, which agrees satisfactorily with the previous analysis of the co-authorship networks. For this reason, it cannot be considered that it is the same transition.

For the curves of the average domain size ($\overline{{S_{d} }}$) as a function of *t*_*s*_, in Fig. 7b), 4 stages can be analyzed. The first corresponds to the first-time steps where great instability is observed. This is due to the fact that the temperature prevents the formation of stable domains due to the low density of links. In terms of co-authorship, this indicates that various working groups are being formed, but strong links have not yet been formed to last over time. Note how the error bars show the great randomness of the links. In the second stage, the average domain size tends to stagger. In particular, at *t*_*p*_ = 31 in two steps. These stable sections were also observed in the co-authorship analysis (see Fig. 6). Apparently, they can occur anywhere on the curve during community size growth. In the third stage, the average domain size increases as time passes; in the middle of this stage it is observed in the graph of the number of domains that *N*_*d*_ tends towards stability, approximately. This fact is a sign that the new spins that are added to existing domains. In the last stage $\overline{{S_{d} }}$ tends to stability. Again, the speed with which the system goes through these 4 stages is higher for distributions with shorter peak times, the result of higher initial densities at lower times.

## Conclusions

A methodology based on Ising-type magnetic models was implemented that made it possible to describe the behavior of dynamic and weighted co-authorship networks. The temporal distribution of new nodes was identified as a key pattern in networks. The results of real co-authorship networks and computational simulations showed a high correspondence. Observables such as internal energy and the number and size of communities or domains, verified the effectiveness of the modeling implemented in both methods. The inflection point of energy as a function of time showed a change of state which requires a minimum density of links. The inflection point of the number of communities showed a shorter time than the transition in energy and the peak times of the distribution of nodes and links. Given these results, it can be highlighted that co-authorship network systems can be predicted following the time trend established by a magnetic exchange interaction model.

The study provides others considerable contributions: First, this goes beyond of the most popular methods for community detection in co-authorship networks^36^, applying a methodology to identify them. Second, this examines the changing features of several social academic networks through time. Here we go beyond the traditional analysis of static networks^37^. It is essential to highlight this because social connections influence the emergence and fall of an academic theme^38^. Thus, for new researchers, it is not only important to identify a topic, but also its current academic social community. Finally, we propose a new method to find the tipping point in social networks taken from the field of magnetism. This contributes to understand the research topic’s momentum improving the researcher's decisions about the time and energy spent in the process.

## Data Availability

The datasets generated during and/or analysed during the current study are available from the corresponding author on reasonable request. OriginPro 8, RStudio and Gephi were used for image processing^39–41^.
